# Distinct Effects of *Escherichia coli,* *Pseudomonas aeruginosa* and *Staphylococcus aureus* Cell Wall Component-Induced Inflammation on the Iron Metabolism of THP-1 Cells

**DOI:** 10.3390/ijms22031497

**Published:** 2021-02-02

**Authors:** Edina Pandur, Kitti Tamási, Ramóna Pap, Gergely Jánosa, Katalin Sipos

**Affiliations:** Department of Pharmaceutical Biology, Faculty of Pharmacy, University of Pécs, Rókus Str. 2, H-7624 Pécs, Hungary; kitti.tamasi@aok.pte.hu (K.T.); pap.ramona@pte.hu (R.P.); janosa.gergely@gytk.pte.hu (G.J.); katalin.sipos@aok.pte.hu (K.S.)

**Keywords:** macrophage, iron, inflammation, cytokine, fractalkine, hepcidin, ferroportin, heme oxygenase

## Abstract

Macrophages are essential immune cells of the innate immune system. They participate in the development and regulation of inflammation. Macrophages play a fundamental role in fighting against bacterial infections by phagocytosis of bacteria, and they also have a specific role in immunomodulation by secreting pro-inflammatory cytokines. In bacterial infection, macrophages decrease the serum iron concentration by removing iron from the blood, acting as one of the most important regulatory cells of iron homeostasis. We examined whether the Gram-positive and Gram-negative cell wall components from various bacterial strains affect the cytokine production and iron transport, storage and utilization of THP-1 monocytes in different ways. We found that *S. aureus* lipoteichoic acid (LTA) was less effective in activating pro-inflammatory cytokine expression that may related to its effect on fractalkine production. LTA-treated cells increased iron uptake through divalent metal transporter-1, but did not elevate the expression of cytosolic and mitochondrial iron storage proteins, suggesting that the cells maintained iron efflux via the ferroportin iron exporter. *E. coli* and *P. aeruginosa* lipopolysaccharides (LPSs) acted similarly on THP-1 cells, but the rates of the alterations of the examined proteins were different. *E. coli* LPS was more effective in increasing the pro-inflammatory cytokine production, meanwhile it caused less dramatic alterations in iron metabolism. *P. aeruginosa* LPS-treated cells produced a smaller amount of pro-inflammatory cytokines, but caused remarkable elevation of both cytosolic and mitochondrial iron storage proteins and intracellular iron content compared to *E. coli* LPS. These results prove that LPS molecules from different bacterial sources alter diverse molecular mechanisms in macrophages that prepossess the outcome of the bacterial infection.

## 1. Introduction

Macrophages are essential immune cells of the innate immune system [1]. They participate in the development and regulation of inflammation [2]. Macrophages play a fundamental role in fighting against bacterial infections by phagocytosis of bacteria, and they also have a specific role in immunomodulation by secreting pro-inflammatory cytokines, e.g., IL-6, IL-1β and TNF-α [3].

Gram-negative and Gram-positive bacteria activate the macrophages via Toll-like receptors (TLRs) [4]. The Gram-negative bacterial cell wall component lipopolysaccharide (LPS) binds to TLR4, while the Gram-positive cell wall polymer lipoteichoic acid (LTA) activates TLR2 [5,6]. Both receptors activate the NFκB signaling pathway and the transcription of pro-inflammatory cytokines [7]. It has been described that LPS and LTA affect the phagocytic activity of macrophages in different ways [8]. Surbatovic et al. revealed that the cytokine profiles of patients with abdominal sepsis are different in the cases of Gram-negative and Gram-positive bacteremia [9], suggesting the important difference in innate immune responses to two types of bacterial infections [10,11].

In bacterial infection, macrophages contribute to iron sequestration and decreasing serum iron concentration, acting as one of the most important regulatory cells of iron homeostasis [12,13]. The reduction of serum iron content is activated by the secretion of hepcidin, the major regulator of iron metabolism [14]. Hepcidin acts through ferroportin (FP), the only known iron exporter [15]. After binding to FP, hepcidin promotes its internalization and degradation and, as a consequence, iron export from the cells is inhibited [16]. Moreover, hepcidin can occlude the FP iron transporter as well, preventing iron export [17]. This alternative mechanism of action of hepcidin on FP gives the opportunity for the cells to generate a faster response to inflammation as well as to resume iron export quickly [18].

Macrophages use divalent metal transporter-1 (DMT-1) for importing ferrous iron from the extracellular space [19]. The imported iron then can be stored in the cytosolic iron storage protein ferritin (FTH), utilized by the cell for synthetic processes in the cytoplasm, or transported into the mitochondria for heme or iron–sulfur cluster synthesis [20]. The mitochondrion also possesses iron storage capacity with mitochondrial ferritin (FTMT), which provides protection against oxidative damage [21].

Bacterial infection-induced inflammation triggers the secretion of fractalkine (FKN) of endothelial cells [22]. FKN acts as a chemokine, recruiting monocytes to the site of infection [23]. Macrophages are also able to synthetize and secrete FKN that acts in autocrine or paracrine ways on the macrophages [24], promoting their survival [25]. It has been described that FKN is implicated in the regulation of iron homeostasis via its receptor, CX3CR1 [26].

In this study, we examined two different types of LPS obtained from *E. coli* and *P. aeruginosa* Gram-negative bacterial strains, and LTA purified from the Gram-positive bacterium *S. aureus* to reveal the differences between their effects on pro-inflammatory cytokine (IL-1β, IL-6 and TNFα) production, on soluble FKN secretion and on the iron transport and storage of THP-1 human monocytes. We found that *E. coli* LPS showed the most powerful effect on pro-inflammatory cytokine and FKN secretions and *P. aeruginosa* LPS was the least effective on IL-6 secretion, while *S. aureus* LTA was found to have almost no effect on TNF-α synthesis. The action of Gram-negative and Gram-positive bacterial cell wall components was time and concentration dependent. Although the two different types of LPS affected the iron content and heme concentration of THP-1 cells similarly, we found fundamental differences between the effects of LTA and LPS treatments on the expression of iron transport and storage proteins. Based on these results, we suppose that not only the type but the source of the bacterial cell wall components are essential in the mechanism of action on macrophages, considering inflammatory as well as iron metabolism regulatory activities.

## 2. Results

### 2.1. Bacterial Cell Wall Components Trigger the Secretion of Pro-Inflammatory Cytokines in THP-1 Cells Differently

Bacterial infections activate different Toll-like receptors (TLRs) on macrophages and regulate the expression of pro-inflammatory cytokines via the NFκB signaling pathway, but it seems that various bacterial strains may influence the production of inflammatory molecules differently [10,11]. We examined the IL-6, IL-1β and TNF-α secretion of THP-1 cells treated with three bacterial cell wall components, mimicking infections. We utilized lipoteichoic acid (LTA) from *S. aureus* and lipopolysaccharide (LPS) from *E. coli* and *P. aeruginosa*.

**Figure 1:**
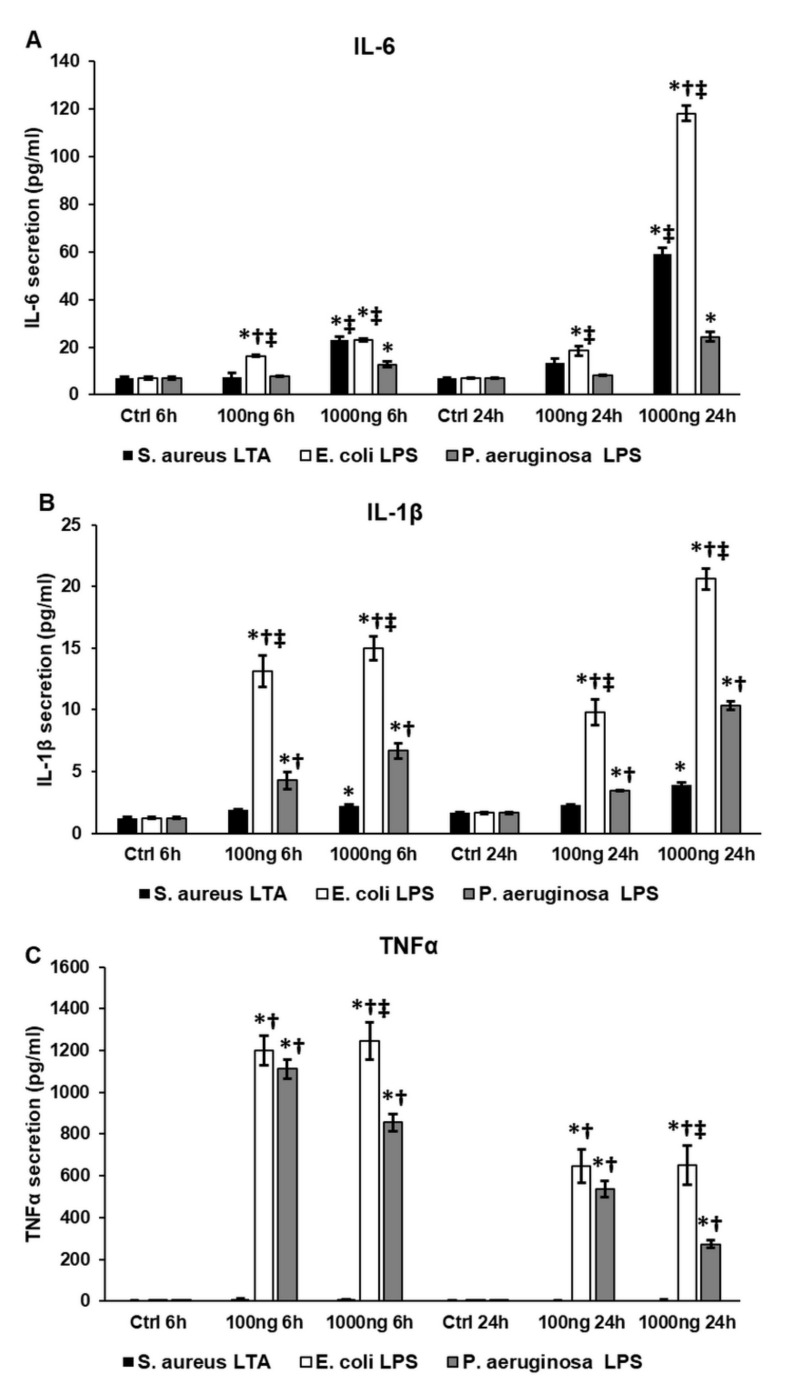
Concentration measurements of pro-inflammatory cytokines of lipopolysaccharide (LPS)- and lipoteichoic acid (LTA)-treated THP-1 cells. Secreted IL-6, IL-1β and TNF-α concentrations of the culture media were determined with human IL-6, IL-1β and TNF-α ELISA kits according to the instructions of the manufacturer. (**A**) Secreted IL-6 concentration of THP-1 cells treated with *S. aureus* LTA, *E. coli* LPS and *P. aeruginosa* LPS. (**B**) IL-1β production of THP-1 cells treated with LTA and LPS isolated from different sources. (**C**) TNF-α secretion of LTA- and LPS-treated THP-1 cells. The columns represent mean values and error bars represent standard deviation (SD) of three independent determinations (*n* = 3). ELISA measurements were carried out in triplicate in each independent experiment. An asterisk * indicates *p* < 0.05 compared to the controls. A cross † indicates *p* < 0.05 compared to LTA treatment and a double cross ‡ shows *p* < 0.05 compared to *P. aeruginosa* LPS treatment.

We found that all three components showed concentration- and time-dependent effects on the pro-inflammatory cytokine secretion of THP-1 cells (Figure 1). THP-1 cells increased both IL-6 and IL-1β production with time, but decreased TNF-α secretion, suggesting a strong and fast TNF-α response at the beginning of the treatments, which was followed by the IL-6 and IL-1β production (Figure 1).

We revealed that *E. coli* LPS was the most powerful activator of pro-inflammatory cytokine secretion at both examined time points (Figure 1A–C) compared to *P. aeruginosa* LPS and *S. aureus* LTA. It seemed that LTA caused a weak inflammatory signal in the THP-1 cells in terms of IL-1β and TNF-α secretion (Figure 1B,C), but was more effective in the case of IL-6 production compared to *P. aeruginosa* LPS (Figure 1A). These results suggest that in the case of Gram-positive infections, IL-6 is probably the major mediator of the inflammation, while Gram-negative bacteria primarily trigger TNF-α secretion.

### 2.2. Lipopolysaccharide (LPS) and Lipoteichoic Acid (LTA) Activate Fractalkine Secretion and CX3CR1 Expression of THP-1 Cells

Fractalkine (FKN) is released by endothelial cells in bacterial infection [22]. FKN is involved in the recruitment of monocytes to the site of infection [23] and binds to its receptor CX3CR1 expressed by macrophages [27]. The CX3CR1 signaling also regulates the NFκB signaling pathway, affecting inflammatory functions of macrophages [24].

**Figure 2:**
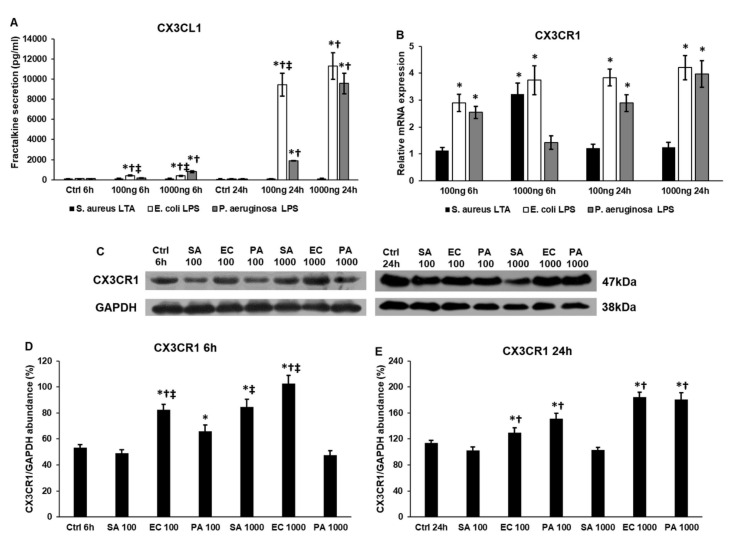
Concentration measurement of fractalkine of lipopolysaccharide (LPS)- and lipoteichoic acid (LTA)-treated THP-1 cells, relative mRNA expression and Western blot analysis of CX3CR1. Secreted fractalkine (FKN) concentrations of the culture media were determined with a human FKN ELISA kit according to the instructions of the manufacturer. Real-time PCR was performed with SYBR Green protocol. β-actin was used as internal control for the normalization. The relative expression of untreated controls was regarded as 1. The mRNA expressions of the treated cells were compared to their appropriate controls (6 h and 24 h). THP-1 cells were collected and pelleted after LPS and LTA treatments. The same amount of protein from each lysate was separated by SDS-PAGE using 12% polyacrylamide gel, and transferred by electroblotting to nitrocellulose membranes. The membranes were probed with anti-CX3CR1 polyclonal rabbit antibodies according to the manufacturer’s protocol. GAPDH was used as loading control. (**A**) Secreted FKN concentration of THP-1 cells treated with *S. aureus* LTA, *E. coli* LPS and *P. aeruginosa* LPS. (**B**) mRNA expression levels of CX3CR1 of THP-1 cells treated with LTA and LPS isolated from different sources. (**C**) Western blot analysis of CX3CR1 protein in LTA- and LPS-treated THP-1 cells. (**D**,**E**) Optical density analyses of CX3CR1 in THP-1 cells. The columns represent mean values and error bars represent standard deviation (SD) of three independent determinations (*n* = 3). ELISA measurements and real-time PCR determinations were carried out in triplicate in each independent experiment. An asterisk * marks *p* < 0.05 compared to the controls. A cross † indicates *p* < 0.05 compared to LTA treatment and a double cross ‡ shows *p* < 0.05 compared to *P. aeruginosa* LPS treatment. Abbreviations: SA: *S. aureus* LTA; PA: *P. aeruginosa* LPS; EC: *E. coli* LPS.

Although LTA treatment increased FKN secretion of the THP-1 cells, the two LPS cell wall components were significantly more effective in triggering FKN production (Figure 2A), suggesting that LPS generates a stronger immune response compared to LTA. We also found that, although at lower concentrations *E. coli* LPS was more effective on the FKN production of THP-1 cells, at a 1000 ng/mL concentration, there was no significant difference between the effects of *E. coli* and *P. aeruginosa* LPS (Figure 2A).

We examined both the mRNA and protein levels of FKN receptor CX3CR1 to see the effect of bacterial cell wall components on the FKN/CX3CR1 interaction. At 6 h, *E.coli* LPS seemed to be the most effective in upregulating CX3CR1 expression (Figure 2B–D). Interestingly, using LTA at higher concentrations at 6 h exerted a significantly higher effect on CX3CR1 level compared to *P. aeruginosa* LPS (Figure 2B–D), suggesting that *S. aureus* LTA was able to alter the FKN/CXC3CR1 interaction as well (Figure 2E). We did not find significant differences between LPS types or between the utilized concentrations at the protein level at 24 h (Figure 2C,E).

### 2.3. Bacterial Cell Wall Components Increase Hepcidin (HAMP) Expression and Hepcidin Secretion of THP-1 Cells

Both inflammation and FKN/CX3CR1 interaction are important inducers of the synthesis of hepcidin, the master iron regulatory peptide hormone [14,26]. Therefore, we examined the mRNA expression of hepcidin (HAMP) and its secretion levels after treatment with different bacterial cell wall components.

**Figure 3:**
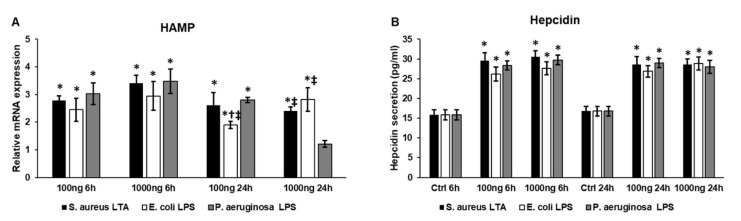
Real-time PCR and ELISA measurements of hepcidin expression in lipopolysaccharide (LPS)- and lipoteichoic acid (LTA)-treated THP-1 cells. Real-time PCR was performed with SYBR Green protocol. β-actin was used as internal control for the normalization. The relative expression of untreated controls was regarded as 1. The mRNA expressions of the treated cells were compared to their appropriate controls (6 h and 24 h). Secreted hepcidin concentrations of the culture media were determined with a human hepcidin ELISA kit according to the instructions of the manufacturer. (**A**) mRNA expression levels of HAMP of THP-1 cells treated with LTA and LPS isolated from different sources. (**B**) Secreted hepcidin concentration of THP-1 cells treated with *S. aureus* LTA, *E. coli* LPS and *P. aeruginosa* LPS. The columns represent mean values and error bars represent standard deviation (SD) of three independent determinations (*n* = 3). Real-time PCR determinations and ELISA measurements were carried out in triplicate in each independent experiment. An asterisk * marks *p* < 0.05 compared to the controls. A cross † indicates *p* < 0.05 compared to LTA treatment and a double cross ‡ shows *p* < 0.05 compared to *P. aeruginosa* LPS treatment.

At the mRNA level, HAMP expression was significantly elevated compared to the control but we did not find a significant difference between the treatments (Figure 3A). Interestingly, at 24 h, 100 ng/mL *E. coli* lipopolysaccharide (LPS) was the less effective on HAMP expression, meanwhile, using 1000 ng/mL of components, *P. aeruginosa* was found to have no effect on HAMP expression (Figure 3A). The action of *S. aureus* lipoteichoic acid (LTA) was similar to *E. coli* in the higher concentration treatments (Figure 3A).

At the protein level, hepcidin levels were similar at 6 h in each treatment (Figure 3B), which were in agreement with the mRNA results (Figure 3A). Interestingly, at 24 h, there was no significant difference among hepcidin levels using different treatments or compared to 6 h levels, suggesting that hepcidin may not be released from the cells into the culture medium but may act inside the cell.

### 2.4. Bacterial Cell Wall Components Alter the Iron Import and Export of THP-1 Cells Differently

Hepcidin affects iron transport by inhibiting iron export via ferroportin (FP) [15,16] and causing iron retention in cells, which decreases the iron content of the extracellular space and inhibits the proliferation of bacteria [12]. We examined the expression of the FP iron exporter and divalent metal transporter-1 (DMT-1) iron importer of the lipoteichoic acid (LTA)- and lipopolysaccharide (LPS)-treated THP-1 cells to reveal the differences between the actions of the three bacterial cell wall components in iron transport.

**Figure 4:**
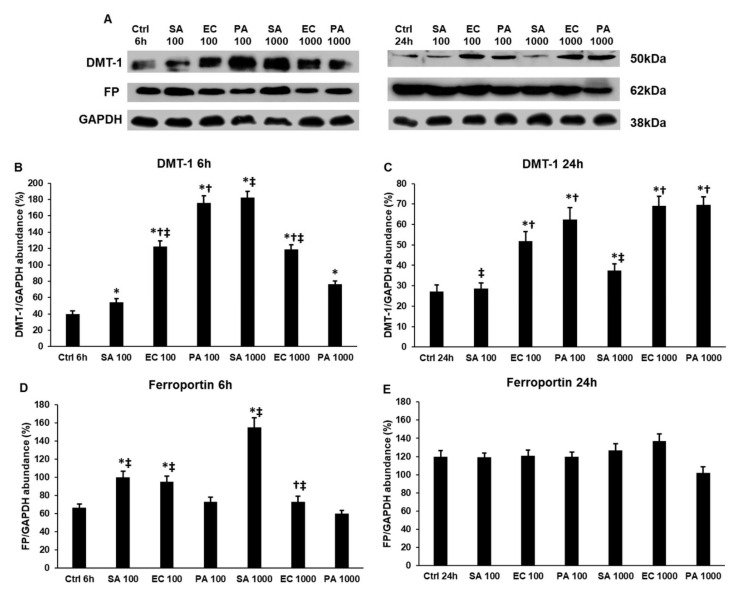
Western blot analyses of iron transporters divalent metal transporter-1 (DMT-1) and ferroportin (FP)in lipoteichoic acid (LTA)- and lipopolysaccharide (LPS)-treated THP-1 cells. THP-1 cells were collected and pelleted after LTA and LPS treatments. The same amount of protein from each lysate was separated by SDS-PAGE using 12% polyacrylamide gel, transferred by electroblotting to nitrocellulose membranes. The membranes were probed with anti-DMT-1 and anti-FP polyclonal rabbit antibodies according to the manufacturer’s protocols. GAPDH was used as loading control. (**A**) Western blot analysis of DMT-1 and FP proteins in THP-1 cells treated with *S. aureus* LTA, *E. coli* LPS and *P. aeruginosa* LPS. (**B**–**C**) Optical density analyses of DMT-1 in THP-1 cells. (**D**,**E**) Optical density analyses of FP in THP-1 cells. The columns represent mean values and error bars represent standard deviation (SD) of three independent determinations (*n* = 3). An asterisk * marks *p* < 0.05 compared to the controls. A cross † indicates *p* < 0.05 compared to *S. aureus* LTA treatment and a double cross ‡ shows *p* < 0.05 compared to *P. aeruginosa* LPS treatment. Abbreviations: SA: *S. aureus* LTA; PA: *P. aeruginosa* LPS; EC: *E. coli* LPS.

Using 100 ng/mL of different cell wall components, the DMT-1 protein level was the highest in the case of *P. aeruginosa* LPS treatment and the lowest level was found in the case of *S. aureus* LTA treatment (Figure 4A,B). We found opposite results using 1000 ng/mL of different cell wall components; LTA treatment caused the highest elevation of DMT-1, while *P. aeruginosa* LPS was the least effective on DMT-1, suggesting that the actions of these components are type and concentration dependent (Figure 4A,B). At 24 h, *S. aureus* LTA did not cause significant change compared to the control and the DMT-1 protein level was still the highest in the case of *P. aeruginosa* LPS treatment using 100 ng/mL of bacterial cell wall components (Figure 4A,C). Treatments with 1000 ng/mL of bacterial cell wall components showed completely different results compared to both the 6 h treatments and to the lower concentration treatments. Both LPS treatments caused significantly higher DMT-1 levels compared to LTA treatment and there was no difference between the two LPS treatments (Figure 4A,C). These results suggest that the iron import into the THP-1 cells during inflammation depends on the type of bacterium, the time of induction and the concentration of the inducer molecule.

Iron export was influenced by LTA and LPS treatments differently, as well. We revealed significant alterations only in 6 h treatments, which raised the possibility that iron export via ferroportin was affected by not only at the expression level but by influencing the export function of FP. *P. aeruginosa* LPS did not alter FP level at all at 6 h or at 24 h (Figure 4A,D,E). The concentration 100 ng/mL of *E. coli* LPS and *S. aureus* LTA increased FP to the same level at 6 h (Figure 4A,D), while when using 1000 ng/mL of cell wall components, only *S. aureus* LTA was able to significantly elevate the FP level (Figure 4A,D). These results suggest that LTA acts differently on iron export compared to the two LPS molecules.

### 2.5. Lipopolysaccharide (LPS) and Lipoteichoic Acid (LTA) Modify the Expression of Cytosolic and Mitochondrial Iron Storage Proteins, Ferritin Heavy Chain (FTH) and Mitochondrial Ferritin (FTMT), Differently

We found that iron transport was modified in the LTA- and LPS-treated THP-1 cells, therefore, we examined whether iron storage was altered as well. We determined the protein levels of the cytosolic iron storage protein FTH and the mitochondrial iron storage protein FTMT to see if the imported iron was stored or was utilized in the cells.

**Figure 5:**
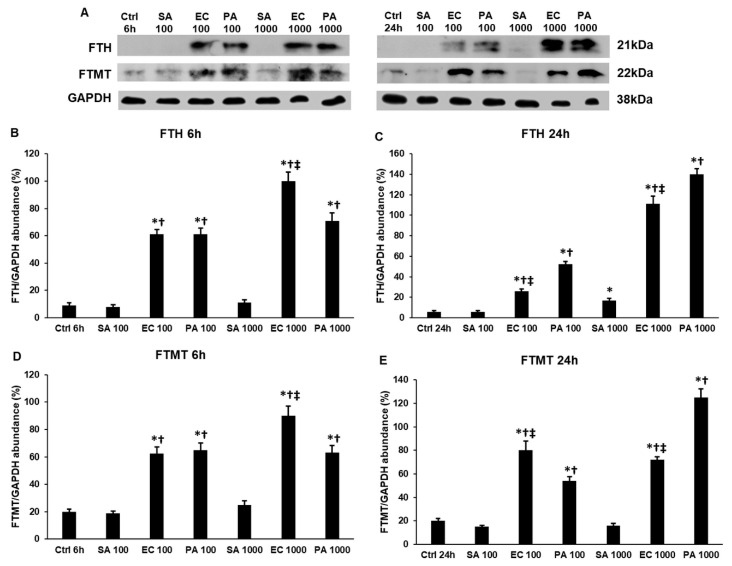
Western blot analyses of iron storage proteins ferritin heavy chain (FTH) and mitochondrial ferritin (FTMT) in lipoteichoic acid (LTA)- and lipopolysaccharide (LPS)-treated THP-1 cells. THP-1 cells were collected and pelleted after treatments with LTA or LPS bacterial cell wall components. The same amount of protein from each lysate was separated by SDS-PAGE using 12% polyacrylamide gel, transferred by electroblotting to nitrocellulose membranes. The membranes were probed with anti-FTH or anti-FTMT polyclonal rabbit antibodies according to the manufacturer’s protocols. GAPDH was used as loading control. (**A**) Western blot analysis of FTH and FTMT proteins in THP-1 cells treated with *S. aureus* LTA, *E. coli* LPS and *P. aeruginosa* LPS. (**B**–**C**) Optical density analyses of FTH in THP-1 cells. (**D**,**E**) Optical density analyses of FTMT in THP-1 cells. The columns represent mean values and error bars represent standard deviation (SD) of three independent determinations (*n* = 3). An asterisk * marks *p* < 0.05 compared to the controls. A cross † indicates p < 0.05 compared to *S. aureus* LTA treatment and a double cross ‡ shows *p* < 0.05 compared to *P. aeruginosa* LPS treatment. Abbreviations: SA: *S. aureus* LTA; PA: *P. aeruginosa* LPS; EC: *E. coli* LPS.

*S. aureus* LTA significantly increased the FTH level only at 24 h (Figure 5A–C), but it did not alter the FTMT level (Figure 5A,D,E), suggesting that iron may be incorporated into enzymes and other proteins, used for heme synthesis or may be released from the cells. In the meantime, both LPS types increased the FTH level at 6 h and their effects increased with concentration (Figure 5A,B). Moreover, the FTH level after *E. coli* LPS treatment was significantly higher compared to *P. aeruginosa*. At 24 h, the opposite effects were observed, *P. aeruginosa*-mediated FTH elevation was significantly higher compared to *E. coli* LPS (Figure 5A,C) at both concentrations (Figure 5A,C).

In the case of FTMT, 6 h and 1000 ng/mL of *E. coli* LPS treatment increased FTMT expression to a significantly higher level compared to *P. aeruginosa* (Figure 5A,D). Interestingly, at 24 h, we found that *E. coli* LPS at a lower concentration exerted a stronger effect on the FTMT protein level compared to *P. aeruginosa* LPS, while, at a higher LPS concentration, the opposite result was revealed: *P. aeruginosa* LPS significantly increased the FTMT level compared to *E. coli* LPS (Figure 5A,E). Based on these observations, we suppose that *P. aeruginosa* and *E. coli* LPS molecules act differently on iron storage, although both increase FTH and FTMT levels. Moreover, the effect of LPS shows concentration dependence.

### 2.6. Bacterial Cell Wall Components Increase Total Iron Content of THP-1 Cells

**Figure 6:**
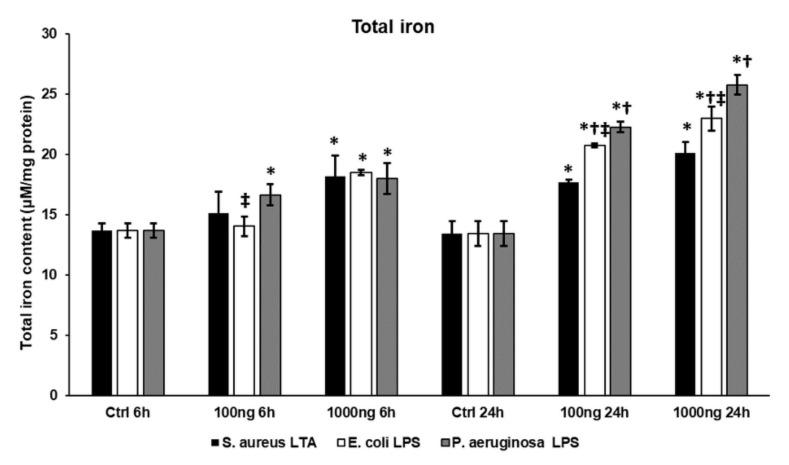
Determinations of total iron content of THP-1 cells treated with *S. aureus* lipoteichoic acid (LTA), *E. coli* lipopolysaccharide (LPS) and *P. aeruginosa* LPS. Iron content of THP-1 cells was determined using a colorimetric ferrozine-based assay and was expressed as µM/mg protein. The columns represent mean values and error bars indicate standard deviation (SD) of three independent determinations (*n* = 3). The measurements were carried out in quadruplicate in each independent experiment. An asterisk * marks *p* < 0.05 compared to the controls. A cross † indicates *p* < 0.05 compared to *S. aureus* LTA treatment and a double cross ‡ shows *p* < 0.05 compared to *P. aeruginosa* LPS treatment.

To prove that treatments with bacterial cell wall components change the iron content of the THP-1 cells, the total intracellular iron content was determined using a ferrozine-based method. Using 100 ng/mL of different bacterial cell wall components, only *P. aeruginosa* lipopolysaccharide (LPS) was able to significantly increase the total iron content at 6 h (Figure 6), while the 1000 ng/mL treatments were all successful in elevating total iron content compared to the control cells. At 24 h, the lipoteichoic acid (LTA) and LPS treatments showed concentration dependence (Figure 6). However, both LPS types significantly increased the intracellular iron content compared to LTA treatment, and *P. aeruginosa* LPS was more effective in elevating iron content compared to *E. coli* LPS at both concentrations (Figure 6). These results suggest that LPS treatments retain iron in the cells in the iron stores, while LTA-treated cells may release it into the extracellular environment.

### 2.7. Lipopolysaccharide (LPS) and Lipoteichoic Acid (LTA) Act Differently on Heme Oxigenase-1 (HO-1) Expression and Heme Concentration of THP-1 Cells

**Figure 7:**
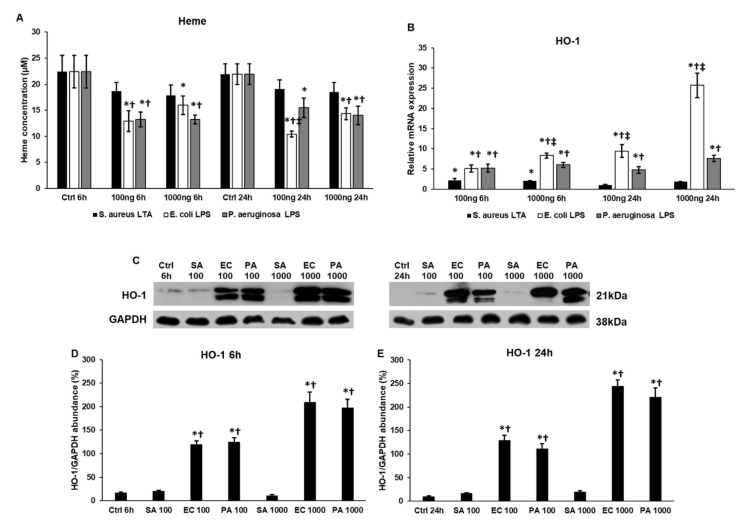
Heme concentration measurements, mRNA and Western blot analyses of heme oxygenase-1 in lipoteichoic acid (LTA)- and lipopolysaccharide (LPS)-treated THP-1 cells. Heme concentration of the treated THP-1 cells was determined using a Heme Assay Kit. Real-time PCR was performed with SYBR Green protocol. β-actin was used as internal control for the normalization. The relative expression of untreated controls was regarded as 1. For the Western blots, the same amount of protein from cell lysates was separated by SDS-PAGE using 12% polyacrylamide gel, transferred by electroblotting to nitrocellulose membranes. The membranes were probed with anti-HO-1 polyclonal rabbit antibody according to the manufacturer’s protocol. GAPDH was used as loading control. (**A**) Heme concentrations of LTA- and LPS-treated THP-1 cells. (**B**) mRNA levels of HO-1 in treated THP-1 cells. (**C**) Western blot analysis of HO-1 protein in THP-1 cells treated with *S. aureus* LTA, *E. coli* LPS and *P. aeruginosa* LPS. (**D**,**E**) Optical density analyses of HO-1 in THP-1 cells. The columns represent mean values and error bars represent standard deviation (SD) of three independent determinations (*n* = 3). Heme measurements and real-time PCR determinations were carried out in triplicate in each independent experiment. An asterisk * marks *p* < 0.05 compared to the controls. A cross † indicates *p* < 0.05 compared to LTA treatment and a double cross ‡ shows *p* < 0.05 compared to *P. aeruginosa* LPS treatment. Abbreviations: SA: *S. aureus* LTA; PA: *P. aeruginosa* LPS; EC: *E. coli* LPS.

Next, we measured the heme concentration of the differently treated cells. We did not find significant alteration in the heme concentration of *S. aureus* LTA-treated THP-1 cells compared to the controls (Figure 7A). Meanwhile, heme concentration significantly decreased with LPS treatments compared to the controls. The action of the two LPS types was similar on heme concentration, a significant difference was only observed at 24 h using 100 ng/mL of LPS; *E. coli* LPS-treated THP-1 cells showed lower heme levels compared to *P. aeruginosa* LPS-treated cells (Figure 7A).

We were interested whether HO-1 enzyme, which is responsible for heme degradation, contributed to the decreasing heme levels of LPS-treated cells. The mRNA expression of HO-1 showed time and concentration dependence in the case of *E. coli* LPS treatments and the same phenomenon was observed at the protein level (Figure 7B–D). These mRNA levels were significantly higher compared to the controls, *P. aeruginosa* LPS and *S. aureus* LTA treatments (Figure 7B). Meanwhile, the HO-1 protein levels were similar after the two types of LPS treatments, but showed significant elevations compared to the controls and LTA treatments (Figure 7C–E). The *S. aureus* LTA treatment exerted a positive effect only at 6 h and only at the mRNA level (Figure 7B). There was no significant change in HO-1 protein level after LTA treatment (Figure 7C–E).

## 3. Discussion

Monocytes/macrophages play a critical role as antigen-presenting immune cells in the case of bacterial infections [28]. Macrophages can recognize bacterial cells via pathogen-associated molecular patterns (PAMPs) and pathogen-induced damage-associated molecular patterns (DAMPs) [29]. Gram-negative and Gram-positive bacterial cells act through different Toll-like receptors (TLRs) of macrophages; the former activates TLR4, while the latter mainly binds to TLR2 [30]. The TLRs can act in MyD88-dependent and -independent ways and activate mitogen-activated phosphorylase kinase (MAPK), NFκB and interferon regulatory factor 3 (IF3) signaling pathways [30]. TLR signaling regulates type I interferon and inflammatory cytokine production (IL-1β, IL-6 and TNFα) of macrophages [28]. Although both the Gram-negative bacterial cell wall component lipopolysaccharide (LPS) and the Gram-positive cell wall component lipoteichoic acid (LTA) activate the same pathways through TLRs, the cytokine profile of the active macrophages may differ [31,32].

It has been suggested that both LPS and LTA obtained from different bacterial strains affect the phagocytic and inflammatory functions of macrophages differently [8,33]. The cause of these alterations has not yet been proven. A possible explanation is modifications in LPS structure [34]. Covalent modifications occur both in the core oligosaccharide and in the lipid A parts, but the structure and composition of the O antigen can also differ within a species [35], or sometimes it is missing [36]. Structural diversity can be found in the case of LTA as well, mainly in the glycolipid anchor residues and in the number of repeating units [37].

In bacterial infection, macrophages contribute to iron sequestration and decrease serum iron concentration, acting as one of the most important regulatory cells of iron homeostasis [12,13]. Previous results have revealed that LPS alters the intracellular iron homeostasis of neurons, microglial cells [38,39], aortic endothelial cells [40], dendritic cells [41] and hepatocytes [42]. In our previous work, we proved that *E. coli* LPS and *S. aureus* LTA acted differently on the iron metabolism of differentiated SH-SY5Y neuronal cells [39] and BV-2 microglia.

According to the literature, it has been revealed that multidrug resistant (MDR) bacteria strains can activate significantly different immune responses [43] and these pathogens can cause a severe inflammatory response as well. The actions of these bacteria may depend on the structure of the bacterial cell wall components, which are recognized by different TLRs [44,45]. The MDR cell wall components activate distinct TLRs [46,47] that can result in the activation of additional signaling pathways, regulating not only cytokine production [43,48] but, for example, transcription factors that are involved in the regulation of iron metabolism as well, e.g., NRF2 that acts as a transcriptional regulator of both iron exporter ferroportin and the oxidative stress-induced protein heme oxygenase 1, which is also responsible for heme degradation [49,50]. Moreover, NRF2 can modify NFκB activity, which results in decreased cytokine production [50,51,52,53]. At the same time, NFκB alters NRF2 synthesis and activity, as well [54].

In this study, we compared the mechanism of action of two types of LPS molecules from *E. coli* and *P. aeruginosa*, and *S. aureus* LTA, on pro-inflammatory cytokine production and on iron transport and storage in the human monocyte/macrophage cell line THP-1. Cytokine production was the highest in the case of *E. coli* LPS treatment and TNF-α secretion was the most prevalent, suggesting that this type of LPS was a strong activator of macrophages. *P. aeruginosa* was less effective on the THP-1 cells and, moreover, LTA-mediated IL-6 production was higher compared to *P. aeruginosa* LPS, suggesting that it mainly affects IL-6 expression, which may contribute to the different effects of LTA on iron metabolism. We also found that LPS exerted an increasing effect on both IL-6 and IL-1β production with time but decreasing action on TNF-α, suggesting that TNF-α may trigger further interleukin production via the activation of MAPK and NFκB signaling pathways [55].

Monocyte recruitment to the site of infection is mediated by fractalkine (FKN), a chemoattractant cytokine expressed and secreted by endothelial cells [56]. The membrane-bound form of FKN activates the adhesion of monocytes via the CX3CR1 receptor to the endothelial cells [57]. Monocytes then migrate to the site of infection/inflammation where they also secrete FKN as a soluble form to regulate action against inflammation [57,58]. The FKN–CX3CR1 interaction activates the intracellular signaling pathways, PLC/PKC, MAPK and NFκB, which influence cell proliferation, apoptosis and inflammatory molecule secretion (FKN, IL-6, IL-1β, TNF-α, etc.) [59]. In our experiments, *S. aureus* LTA treatment resulted in the lowest secretion level of FKN, and CX3CR1 mRNA expression increased only at 6 h using 1000 ng/mL LTA that was correlated with the CX3CR1 protein level. This alteration may contribute to the elevated IL-6 and IL-1β levels. The two types of LPS acted in a concentration- and time-dependent manner on the THP-1 cells, although *E. coli* LPS treatment was more effective in increasing both FKN and CX3CR1 protein levels. These alterations were positively correlated with higher pro-inflammatory cytokine production.

Inflammatory mediators, e.g., IL-6, are strong activators of hepcidin, the iron regulatory hormone, that can modify iron transport and, as a consequence, overall cellular iron metabolism [14,15,16]. Hepcidin is translated as preprohepcidin, and the pre-part is cleaved in the endoplasmic reticulum to form prohepcidin. Then the prohepcidin undergoes proteolytic cleavage and mature hepcidin is secreted from the cells [60]. Stimulation of macrophages with IL-6 activates hepcidin production and causes iron retention in the cells [61]. It has been reported that FKN enhances hepcidin synthesis via CX3CR1 [26]. In our experiments, the mRNA levels of hepcidin (HAMP) were significantly elevated, suggesting that the inflammatory mediators activated hepcidin transcription. At the protein level, there was no significant difference between the effects of different bacterial cell wall components; the THP-1 cells secreted a similar amount of hepcidin into the culture medium. However, we cannot exclude that hepcidin may remain intracellular as prohepcidin and act inside the cells [62].

Hepcidin causes iron retention in the cells by regulating iron efflux via the iron exporter ferroportin (FP) [63]. Hepcidin can bind to FP, activating its internalization and degradation, but, as an alternative mechanism, of action hepcidin can occlude the FP channel as well, inhibiting iron export from the cells [17,18]. We found that only *S. aureus* LTA significantly increased the FP protein level at 6 h at both concentrations, while at 24 h, there were no alterations in FP levels compared to the controls. These results suggest that *S. aureus* LTA treatment may not inhibit iron release from the cells. Moreover, it seems that FP internalization was not activated by bacterial cell wall components, which suggests that hepcidin may physically inhibit iron release from the cells.

Iron uptake of the cells was also affected by the bacterial cell wall components. The divalent metal transporter-1 (DMT-1) protein, expressed by both the plasma membrane and the endosome membrane, has a dual role in iron uptake into the cells and the release of iron from the endosome [64]. The DMT-1 protein level was elevated in LTA-treated cells at 6 h and, moreover, this treatment was the most effective on DMT-1 levels using the higher LTA concentration. Although we found increasing DMT-1 levels with 100 ng/mL treatments (SA-EC-PA), the opposite results were observed using 1000 ng/mL, and the levels decreased in the same order (SA-EC-PA), suggesting that the effects of the examined bacterial cell wall components were type and concentration dependent. Later, the DMT-1 expression elevation mediated by LTA was less effective, but the two LPS molecules were able to maintain a high DMT-1 level, suggesting that LTA generated a fast response in iron uptake, while LPS molecules were more effective later on.

Iron accumulation induces the expression of the iron storage protein ferritin heavy chain (FTH), while the mitochondrial iron storage protein seems to act as an antioxidant molecule preventing reactive oxygen species (ROS) generation [65,66]. The determination of the levels of the cytosolic iron storage protein FTH and mitochondrial iron storage protein mitochondrial ferritin (FTMT) supports our hypothesis that *S. aureus* LTA-treated THP-1 cells do not store iron, but release it after import; neither FTH nor FTMT showed alterations at the protein level. Comparing *E. coli* and *P. aeruginosa* LPS treatments *E. coli* LPS increased both FTH and FTMT levels more efficiently at 6 h but, later, *P. aeruginosa* was more effective in elevating the levels of iron storage proteins. These results suggest that the two LPS molecules act similarly on iron storage but in different time periods. The total iron content of *S. aureus*-treated cells was the lowest when comparing the three treatments, although it was still significantly higher compared to the control THP-1 cells. Based on the iron storage protein levels, it seems that, in LTA-treated cells, the iron is stored in the labile iron pool or incorporated into other types of proteins, e.g., peroxidases, which protect against iron-mediated cytotoxicity. LPS increased the total iron content, which was in accordance with the levels of iron storage proteins; the *P. aeruginosa*-treated THP-1 cells showed significantly higher total iron content compared to the *E. coli* LPS-treated cells.

It has been described that LPS induces heme oxygenase-1 (HO-1) expression in monocytes [67] and may contribute to the downregulation of ROS production mediated by heme–oxygen interaction [68]. Moreover, it has been observed that the cytoprotective effect of HO-1 requires the expression of FTH [69]. *S. aureus* LTA treatment did not cause significant change in heme concentration, which was in accordance with the unaltered HO-1 protein level. However, there was no significant alteration between the effects of the two LPS types, as both of them decreased heme concentration and increased HO-1 mRNA and protein levels, and it seems that these changes may provide a cytoprotective effect.

HO-1 provides an anti-inflammatory effect as well, and it is activated by the NRF2 transcription factor, and regulated by inflammatory processes [70]. HO-1 releases CO by the degradation of heme, which attenuates inflammation [71]. Moreover, NRF2 is regulated by the NFκB signaling pathway, which is one of the downstream pathways of CX3CR1 [59,71], controlling pro-inflammatory cytokine production. Other pathways, e.g., MAPK and PKC regulated by FKN/CX3CR1, are also implicated in the upregulation of HO-1 [59].

*S. aureus* LTA was less effective in activating pro-inflammatory cytokine expression, which may be related to the low production of FKN, which is the activator of CX3CR1. We did not find upregulation of CX3CR1 in the LTA-treated THP-1 cells, which could be the reason for the unchanged FTH and HO-1 expression. LTA-treated cells increased iron uptake but did not store iron, suggesting that the cells maintained iron efflux via FP. This molecular evidence may be the underlying cause of the slower clinical onset of Gram-positive bacterial infections [9].

*E. coli* and *P. aeruginosa* LPS acted similarly on THP-1 cells, but the rates of the alterations of the examined proteins were different. *E. coli* LPS was more effective in increasing pro-inflammatory cytokine production, and we found a robust and fast response to the presence of LPS. Meanwhile, *E. coli* LPS caused less dramatic alterations in iron metabolism. *P. aeruginosa* LPS-treated cells produced smaller amounts of pro-inflammatory cytokines, but caused remarkable elevation of both cytosolic and mitochondrial iron storage proteins and intracellular iron content compared to *E. coli* LPS, proving that LPS molecules from different bacterial sources alter diverse molecular mechanisms in macrophages that prepossess the outcome of the bacterial infection. Taken together, we propose that the type of the bacterium determines how the iron metabolism changes in infection. These alterations may affect and modify the diagnostic methods and the therapeutic approach. Based on the results, the serum pro-inflammatory cytokine measurements, especially the TNF-α concentration, could be used as an early prognostic factor for Gram-negative bacterial infection as well as the serum iron concentration.

## 4. Materials and Methods

### 4.1. Cell Culture and Treatments

THP-1 human monocyte suspension cell line (ATCC TIB-202) was maintained in Roswell Park Memorial Institute (RPMI) 1640 Medium (Lonza Ltd., Basel, Switzerland) supplemented with 10% fetal bovine serum (FBS, Biowest, Nuaillé, France) and 1% penicillin–streptomycin (P/S, Lonza Ltd., Basel, Switzerland). The Gram-negative cell wall components (LPS) obtained from *E. coli* and from *P. aeruginosa* were purified with phenol extraction (*E. coli 055:B5*, *P. aeruginosa 10*, Sigma-Aldrich Kft., Budapest, Hungary). The Gram-positive cell wall polymer LTA was purified from *S. aureus* (Sigma-Aldrich Kft., Budapest, Hungary). LPS and LTA stock solutions (1 mg/mL) were made in distilled water. The experiments were carried out in a humidified atmosphere containing 5% CO_2_ at 37 °C. For each treatment, 4 × 10^5^ cells were plated onto 6-well plates suitable for suspension culture (Sarstedt Kft., Budapest, Hungary) and were cultured for 24 h before the treatments. The cells were treated with 10, 50, 100, 500 and 1000 ng/mL *E. coli* or *P. aeruginosa* LPS or *S. aureus* LTA for 6 h, 24 h and 48 h. Concentrations and time durations of LPS and LTA treatments were selected according to these time and concentration dependence analyses; 100 and 1000 ng/mL LPS and LTA concentrations and 6 h and 24 h long treatments were chosen for the experiments based on the pro-inflammatory cytokine production (Supplementary Figure S1). Untreated cells were used as controls.

### 4.2. RNA Isolation, cDNA Synthesis and Quantitative Real-Time PCR

THP-1 cells were seeded onto 6-well culture dishes at a density of 4 × 10^5^ cells/well and were cultured for 24 h before the treatments. After the treatments, cell cultures were collected in sterile tubes by centrifugation at 1200 rpm. The cell pellets were washed with phosphate-buffered saline (PBS, Lonza Ltd., Basel Switzerland). Total RNA was isolated using the Quick RNA MiniPerp Kit (Zymo Research, Irvine, CA, USA). RNA concentration of the samples was determined using a MultiSkan GO spectrophotometer (Thermo Fisher Scientific Inc., Waltham, MA, USA) and the RNA measurement protocol of SkanIt Microplate Reader Software (Thermo Fisher Scientific Inc., Waltham, MA, USA). RNA samples were reverse transcribed to cDNA from 200 ng of total RNA using the iScript cDNA synthesis kit (Bio-Rad Laboratories, Hercules, CA, USA) according to the manufacturer’s protocol. Quantitative real-time PCR analysis was performed using gene-specific primers in a CFX96 Real-Time PCR Detection System (Bio-Rad Laboratories, Hercules, CA, USA) using iTaq Universal SYBR Green Supermix (Bio-Rad Laboratories, Hercules, CA, USA) in 20 µL of total volume. Data were analyzed with CFX Maestro 1.1 Software (Bio-Rad Laboratories, Hercules, CA, USA) using the comparative 2^ΔΔ^Ct (Livak) method. For normalization, we used β-actin as a housekeeping gene in each experiment, which was advised by CFX Maestro 1.1 Software. Relative expression of the controls was regarded as 1. We used 6 h, 24 h and 48 h untreated cells as controls of the treated cells, respectively. The mRNA expression of the treated cells was compared to the appropriate controls. Real-time PCR determinations were carried out in triplicate in each independent experiment. Nucleotide sequences of the primers used in the experiments are described in Table 1.

### 4.3. Enzyme-Linked Immunosorbent Assay (ELISA) Measurements

After each treatment, supernatants of treated and control THP-1 cells were collected and stored at −80 °C until the measurements. The secreted IL-6, IL-1β and TNF-α contents of the culture media were determined with IL-6, IL-1β and TNF-α human ELISA kits (Thermo Fisher Scientific Inc., Waltham, MA, USA) according to the instructions of the manufacturer. The secreted mature hepcidin content of the samples was determined with a Human Hepcidin Quantikine ELISA Kit (Bio-Techne R&D Systems Kft., Budapest, Hungary). The secreted fractalkine protein concentration was determined with a Human Fractalkine ELISA Kit (Wuhan Fine Biotech Co., Ltd., Wuhan, China). All measurements were performed in triplicate according to the protocols of the manufacturers.

### 4.4. Western Blotting

The cells were collected by centrifugation after each treatment. Pelleted cells of each sample were lysed with 200 µL of lysis buffer (50 mM Tris-HCl, pH 7.4, 150 mM NaCl, 0.5% Triton-X 100) supplemented with a complete mini protease inhibitor cocktail (Roche Ltd., Basel, Switzerland). Protein contents of the samples were determined with a DC Protein Assay Kit (Bio-Rad Laboratories, Hercules, CA, USA). The same amount of protein from each sample was loaded onto 12% polyacrylamide gel. The Bio-Rad Mini Protean Tetra Cell (Bio-Rad Laboratories, Hercules, CA, USA) was used for electrophoresis. The gels were transferred by electroblotting to nitrocellulose membranes (Pall AG, Basel, Switzerland). The membranes were blocked with blocking solution containing 5% (*w*/*v*) non-fat dry milk (Bio-Rad Laboratories., Hercules, CA, USA) for 1 h at room temperature with gentle shaking. The membranes were incubated with the following polyclonal rabbit antibodies for 1 h at room temperature in the case of anti-fractalkine receptor IgG (1:1000; Wuhan Fine Biotech Co., Ltd., Wuhan, China), anti-ferroportin IgG (1:1000; Bio-Techne, Minneapolis, MN, USA), anti-DMT-1 (Thermo Fisher Scientific Inc., Waltham, MA, USA) and anti-mitochondrial ferritin (FTMT) (1:1000, Thermo Fisher Scientific Inc., Waltham, MA, USA) and for overnight at 4 °C in the case of anti-FTH IgG (1:1000; Cell Signaling Technology Europe, Leiden, the Netherlands) and anti-HO-1 IgG (1:1000; Cell Signaling Technology Europe, Leiden, the Netherlands). GAPDH (1:3000; Merck KGaA, Darmstadt, Germany) was used as loading control of the Western blots. We used horseradish peroxidase (HRP)-linked goat anti-rabbit IgG as a secondary antibody (1:2000; Cell Signaling Technology Europe, Leiden, the Netherlands) for 1 h at room temperature. We used traditional colorimetric detection using Fuji medical X-ray film (Fujifilm Corporation, Tokyo, Japan). Protein detection was carried out using WesternBright ECL chemiluminescent substrate (Advansta Inc., San Jose, CA, USA). Optical density was determined using ImageJ software [72], and was expressed as a percentage of target protein/GAPDH abundance.

### 4.5. Total Iron Content Measurements

THP-1 cells were collected after treatment by centrifugation. The pelleted cells were lysed with 200 µL of 50 mM NaOH at room temperature for 2 h with gentle shaking (125 rpm). Neutralization was carried out by adding 100 µL of 10 mM HCl to 100 µL of sample. After neutralization, the samples were mixed with 100 µL of iron-releasing reagent (1.4 M HCl, 4.5% (*w*/*v*) KMnO_4_ in H_2_O) and were incubated for 2 h at 60 °C. After iron release from proteins, 30 µL of iron detection reagent (6.5 mM ferrozine; 6.5 mM neocuproine; 2.5 M ammonium acetate; 1 M ascorbic acid) were added to each tube. Following incubation at room temperature for 30 min, the absorbance was measured at 550 nm using a MultiSkan GO spectrophotometer (Thermo Fisher Scientific Inc., Waltham, MA, USA). The iron content was determined by an FeCl_3_ (0–300 µM) standard curve [73]. Protein concentration of the samples was measured with a DC Protein Assay Kit (Bio-Rad Laboratories, Hercules, CA, USA). The iron content of each sample was normalized to the protein content and was expressed as µM iron/mg protein. Intracellular total iron measurements were carried out in quadruplicate in each independent experiment.

### 4.6. Heme Concentration Determination

THP-1 cells were collected after treatment by centrifugation. The heme concentration was determined using a Heme Assay Kit (Sigma-Aldrich Kft. Budapest, Hungary). The cell pellets were lysed with 100 µL of ultrapure water at room temperature for 15 min with shaking (650 rpm). Then 50 µL of each sample were mixed with 200 µL of Heme Reagent and were incubated at room temperature for 5 min. The absorbance was measured at 400 nm using a MultiSkan GO spectrophotometer (Thermo Fisher Scientific Inc., Waltham, MA, USA). Heme concentration was calculated according to the instructions of the manufacturer. The heme concentration was expressed as µM. Heme concentration determinations were carried out in quadruplicate in each independent experiment.

### 4.7. Statistical Analysis

For all data, n corresponds to the number of independent experiments. ELISA measurements and real-time PCR determinations were carried out in triplicate in each independent experiment. The intracellular total iron measurements and heme concentration determinations were carried out in quadruplicate in each independent experiment. Western blots are representative of three independent experiments. Statistical analysis was performed using SPSS software (IBM Corporation, Armonk, NY, USA). Statistical significance was determined by one-way ANOVA followed by Tukey’s HSD post hoc test. Data are shown as mean ± standard deviation (SD). Statistical significance was determined at *p* value < 0.05.

## Figures and Tables

**Table 1:** Real-time PCR gene primer list.

Primer	Sequence 5’ → 3’
HAMP forward	CAGCTGGATGCCCATGTT
HAMP reverse	TGCAGCACATCCCACATC
Fractalkine receptor forward	CCATTAGTCTGGGCGTCTGG
Fractalkine receptor reverse	GTCACCCAGACACTCGTTGT
HO-1 forward	ACCCATGACACCAAGGACCA
HO-1 reverse	ATGCCTGCATTCACATGGCA
β-actin forward	AGAAAATCTGGCACCACACC
β-actin reverse	GGGGTGTTGAAGGTCTCAAA

## Data Availability

Not applicable.

## Supplementary Materials

The following are available online at https://www.mdpi.com/1422-0067/22/3/1497/s1.

## Abbreviations

CX3CL1	fractalkine
CX3CR1	fractalkine receptor
DAMP	pathogen-induced damage-associated molecular pattern
DMT-1	divalent metal transporter-1
EC	Escherichia coli
FKN	fractalkine
FP	ferroportin
FTH	ferritin heavy chain
FTMT	mitochondrial ferritin
GAPDH	glyceraldehyde 3-phosphate dehydrogenase
HAMP	hepcidin antimicrobial protein
HO-1	heme oxygenase-1
IF3	interferon regulatory factor 3
IL-1β	Interleukin-1β
IL-6	Interleukin-6
LPS	lipopolysaccharide
LTA	lipoteichoic acid
MAPK	mitogen activated phosphorylase kinase
NFκB	nuclear factor kappa-light-chain-enhancer of activated B cells
NRF2	nuclear factor erythroid 2-related factor 2
PA	Pseudomonas aeruginosa
PAMP	pathogen-associated molecular pattern
PKC	protein kinase C
PLC	phospholipase C
ROS	reactive oxygen species
SA	Staphylococcus aureus
TLR	Toll-like receptor
TNFα	tumor necrosis factor α
