# Supplementary material for: Distinct Effects of Escherichia coli, Pseudomonas aeruginosa and Staphylococcus aureus Cell Wall Component-Induced Inflammation on the Iron Metabolism of THP-1 Cells
Source: Int J Mol Sci. 2021 Feb 2;22(3):1497. doi: 10.3390/ijms22031497 (PMC7867333; doi:10.3390/ijms22031497)
Supplement: Supplementary file 1 [file ijms-22-01497-s001.pdf]

Supplementary materials

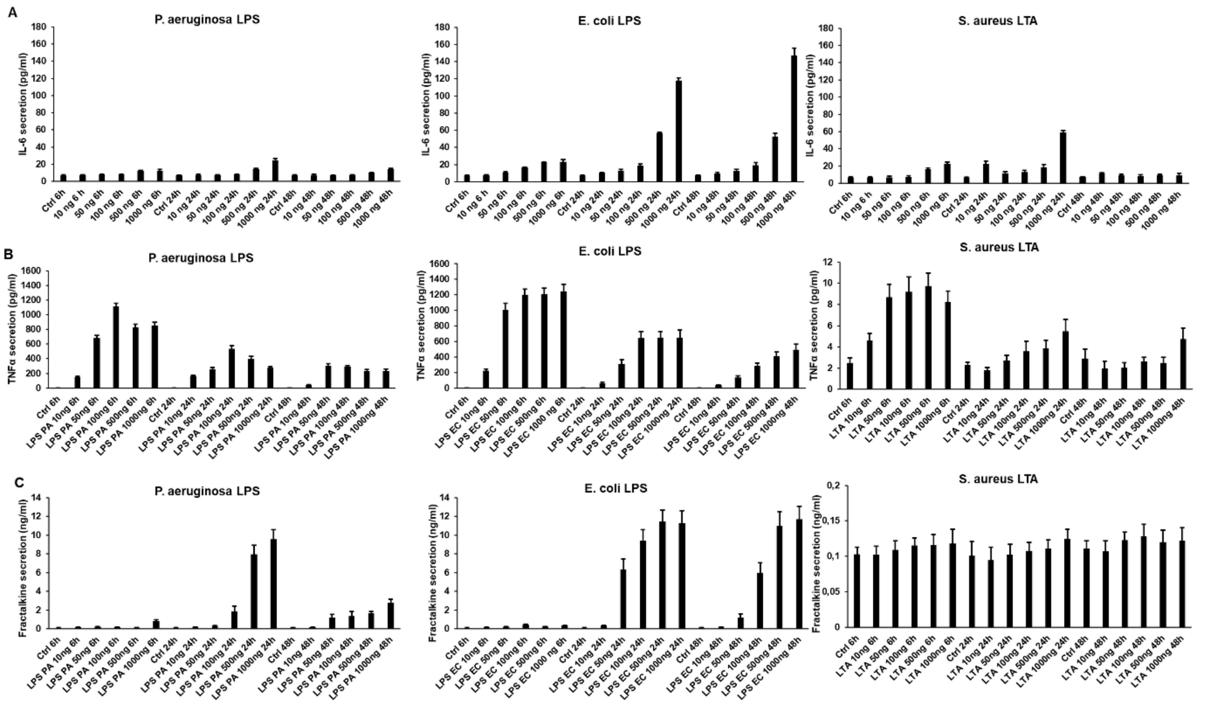

Figure 1. IL-6, TNFα and FKN ELISA measurements from the supernatants of *E. coli* and *P. aeruginosa* lipopolysaccharide (LPS), and *S. aureus* lipoteichoic acid (LTA) treated THP-1 cells. ELISA measurements were carried out according to the protocols of the manufacturers. All measurements were performed in triplicate in each independent experiments. The columns represent mean values and error bars represent standard deviation (SD) of three independent determinations (n = 3). Concentrations and time durations of LPS and LTA treatments were selected according to these time and concentration dependence analyses.
